# Songwriting Group Music Therapy to promote psychological adjustment in informal caregivers of elderly people with dependency: a mixed methods study

**DOI:** 10.3389/fpsyg.2024.1334875

**Published:** 2024-03-06

**Authors:** Paula Pérez-Núñez, Clare O’Callaghan, Juan Francisco López-Paz, Aitana Ruiz de Lazcano, Alicia Aurora Rodríguez, Imanol Amayra

**Affiliations:** ^1^Faculty of Health Sciences, Department of Psychology, University of Deusto, Bilbao, Spain; ^2^Department of Medicine, St. Vincent's Hospital, The University of Melbourne, Melbourne, VIC, Australia; ^3^Caritas Christi (Palliative Care Unit) and Psychosocial Cancer Care, St. Vincent’s Hospital, Melbourne, VIC, Australia

**Keywords:** informal caregivers, mixed methods, quality of life, psychological adjustment, Songwriting Group Music Therapy

## Abstract

**Introduction:**

Informal caregivers of elderly people with dependency (EPD) provide intensive care that can affect their quality of life (QoL). Psychosocial interventions such as music therapy are important to work on their self-care. The aim of this study is to analyze, with a mixed method approach, the experience of participating in a Songwriting Group Music Therapy (SGMT) intervention on informal caregivers of EPD.

**Methods:**

A total of 11 groups, with a convenience sample of 61 caregivers, received 10 SGMT sessions. Quantitative information related to QoL variables (anxiety, depression, spirituality, burden, and coping) was collected before and after the intervention and at 3 months of follow-up. Regarding qualitative data, an open-ended question about the experience of participating was asked.

**Results:**

Significant changes were shown, sustained over time, in trait anxiety and depression and subscales including inner peace, social functioning, and mental health. Three themes were generated from the thematic analysis, including that SGMT participation can enhance personal growth, bring out and enable work on emotions, and promote helpful interpersonal dynamics.

**Discussion:**

The findings indicate that SGMT is a useful intervention for informal caregivers of EPD, promoting psychological adjustment, enhanced coping, emotional regulation, and social support. This study reinforces the findings with caregivers of other populations, providing new results and highlighting the benefits of SGMT for caregivers of EPD.

## Introduction

Due to the aging of the population, there has been an increase in the number of elderly people with dependency (EPD) who require health and social resources involving all care areas (WHO, 2011). Dependency can be described as the difficulty in performing activities of daily living autonomously, requiring the help of a third person (Salvador-Carulla et al., 2010; Diyanto et al., 2016), being an impact factor in the quality of life (QoL) not only for the EPD but also for the caregiver (Millán-Calenti et al., 2010). Therefore, the care provided to EPD extends beyond the patient to support families and relatives due to the emotional impact of the situation (Belletti et al., 2011). It is within this context that the figure of the main informal caregiver appears, defined as the person who assumes the responsibility of helping with the basic and instrumental needs of the dependent family member’s daily life, without financial remuneration (Goodhead and Mcdonald, 2007; López Gil et al., 2009). The burden of caregiving can be divided into objective burden, referring to the economic impact and the time spent on daily care, and subjective burden related to the emotional responses arising from caregiving (Alves et al., 2019).

Adopting the role of informal caregiver is a major and usually stressful change in one’s lifestyle, which can lead to negative impacts on health and QoL. Caregiving has been linked to worsening of the caregiver’s physical and mental health (Schulz et al., 1990, 1995, 1997; Pot et al., 1997; Pinquart and Sörensen, 2003; Verbakel et al., 2016; Bom et al., 2018). Symptomatology often includes depression, anxiety, sleep disturbances, fatigue, and impaired social relationships (Espinoza-Salgado et al., 2017; Choi and Seo, 2019; Perpiñá-Galvañ et al., 2019; Oechsle et al., 2020). Prolonged exposure to caregiving can result in the persistence of these symptoms, potentially leading individuals to experience burnout (De Valle-Alonso et al., 2015). The risk factors that heighten the probability of suffering from this syndrome are related to the greater needs of the care receiver, being a spouse, when caregivers are more cognitively impaired, have more physical symptoms, and are at risk for clinical depression (Beach et al., 2005). Studies, therefore, need to examine potential support interventions for informal caregivers, including those aimed at promoting self-care (Dueñas Granados, 2022).

One of those supports is the psychosocial interventions, which is focused on psychological or social actions that generate changes in psychological, social, biological, and/or functional outcomes, with an emphasis on the construction of mediators (England et al., 2015). These types of interventions allow caregivers’ self-care and could focus on strengthening resilience, supporting spiritual well-being, self-compassion, and appropriate coping strategies as mediators to improve QoL and well-being (Giesbrecht et al., 2015; Weisser et al., 2015; Espinoza-Salgado et al., 2017). Advances in psychosocial interventions are a very important part of care to provide a more holistic intervention (Warth et al., 2019; Ahn et al., 2020). One non-pharmacological treatment and complementary therapy based on creative arts is music therapy (Belletti et al., 2011; Warth et al., 2019; Kim et al., 2020). The World Federation of Music Therapy (WFMT, 2011) defines it as the use of music and/or its elements by a qualified music therapist, with a patient or group, in a process intended to facilitate and promote relevant therapeutic goals, in order to assist physical, psychological, social, and cognitive needs. Although there are different approaches to understanding music therapy, there are certain similarities in the different definitions, mainly the presence of three indispensable elements: the music, the patient, and the music therapist (Wigram et al., 2002).

The objectives of music therapy are flexible, adapting to the needs of individuals, and having in common the improvement of their well-being. One of the techniques in music therapy that allows one to achieve these objectives is therapeutic songwriting, which is defined as “the process of creating, notating, and/or recording lyrics and music within a therapeutic relationship to address psychosocial, emotional, cognitive, and communicative needs of a client or group of clients” (Baker, 2015). Songs are a way through which you can explore emotions, expressing who you are and how you feel, and thus, songwriting presents several strengths (Baker and Wigram, 2005). In this sense, after an inductive analysis, Baker (Baker, 2015), affirmed that this technique involves a therapeutic process, being a medium for emotional expression and social activity while transforming the songwriter’s environment.

Considering that the needs of informal caregivers are mainly focused on emotional and social dimensions, songwriting is a very appropriate technique. In the few existing studies about music therapy and informal caregivers, songwriting is the most used technique. The studies that focused on the applications of music therapy in group formats with informal caregivers have shown a positive impact on QoL (Clair et al., 1993; Clair, 2002; Brotons and Marti, 2003; Magill, 2009; Choi, 2010; Kim and Dvorak, 2018; Potvin et al., 2018; Gok Ugur et al., 2019; Clark et al., 2020; García-Valverde et al., 2020; Valero-Cantero et al., 2020; Wilhelm and Wilhelm, 2020; Brault and Vaillancourt, 2022; Denk, 2023; Whitford et al., 2023). However, most studies focus on the intervention of the caregiver and cared-for person dyad. Focus on music therapy interventions for caregivers only is an important gap because informal caregivers have individual needs outside of caregiving, and this means that self-care and caregiving overload are not addressed. Furthermore, music therapy caregiver research tends to focus only on caregivers of people with dementia or receiving palliative care (Steiner-Brett, 2023). Given the positive findings from these investigations in various domains, it is worthwhile to investigate the potential impact of music therapy on informal caregivers of EPD, as there is currently a lack of research in this specific area. Furthermore, to truly understand the benefits of music therapy for caregivers, it is relevant to include not only quantitative information but also the perception of the participants as a necessity in the evaluation of health outcomes. Quantitative research uses close-ended researcher-created measures to examine what is important to researchers, whereas qualitative research is also needed to understand what participants consider important (Steiner-Brett, 2023).

This study aims to analyze, with a mixed method approach, the experience of participating in a Songwriting Group Music Therapy (SGMT) intervention on informal caregivers of EPD.

The specific objectives are:

- to examine the perception of SGMT experiences of informal caregivers of EPD.
- to analyze the changes in caregivers’ anxiety, depression, burden, perceived health, spirituality, and coping strategies before, immediately following and after 3 months of the SGMT intervention.
- to determine and understand the impact of the SGMT intervention on the QoL of caregivers and their caregiving situation.

## Methods

### Design

**Figure 1:**
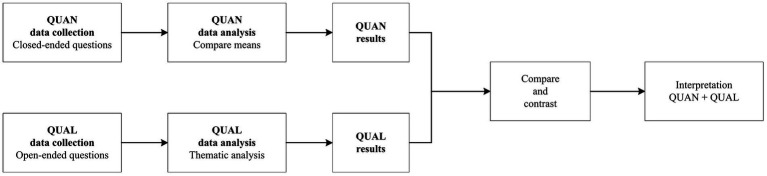
Convergent mixed methods design. QUAN, quantitative; QUAL, qualitative. Adapted from Creswell and Plano Clark (2007).

A mixed methods design was used to analyze caregivers’ experience of participating in a 10-session SGMT program while caring for EPD. Mixed methods research aligns with the pragmatic worldview which is not committed to one system of philosophy or reality and is real-world practice-oriented (Creswell and Creswell, 2018). The mixed method type used was the convergent design, through which quantitative and qualitative data were collected concurrently, and the findings were then converged (Figure 1) (Creswell and Creswell, 2018). Close-ended and open-ended questions were used to deeply explore caregivers’ music therapy experience.

### Participants

A convenience sampling method was carried out. Caregivers were contacted through the different municipal services and programs offered to support the needs of informal caregivers in Bizkaia, Spain. Participants were included in the sample if they met the following criteria: (a) the main informal caregiver of an elderly dependant; (b) the dependant person was older than 60 years; (c) at least 18 years of age; and (d) signed the informed consent form, accepting the assessment and intervention. Exclusion criteria were: as follows (a) not residing in Spain; (b) younger than 18 years; (c) had uncompensated sensory and/or cognitive deficits that prevented response to the assessment; (d) illiteracy; and (e) diagnosis of any severe or serious mental illness.

**Figure 2:**
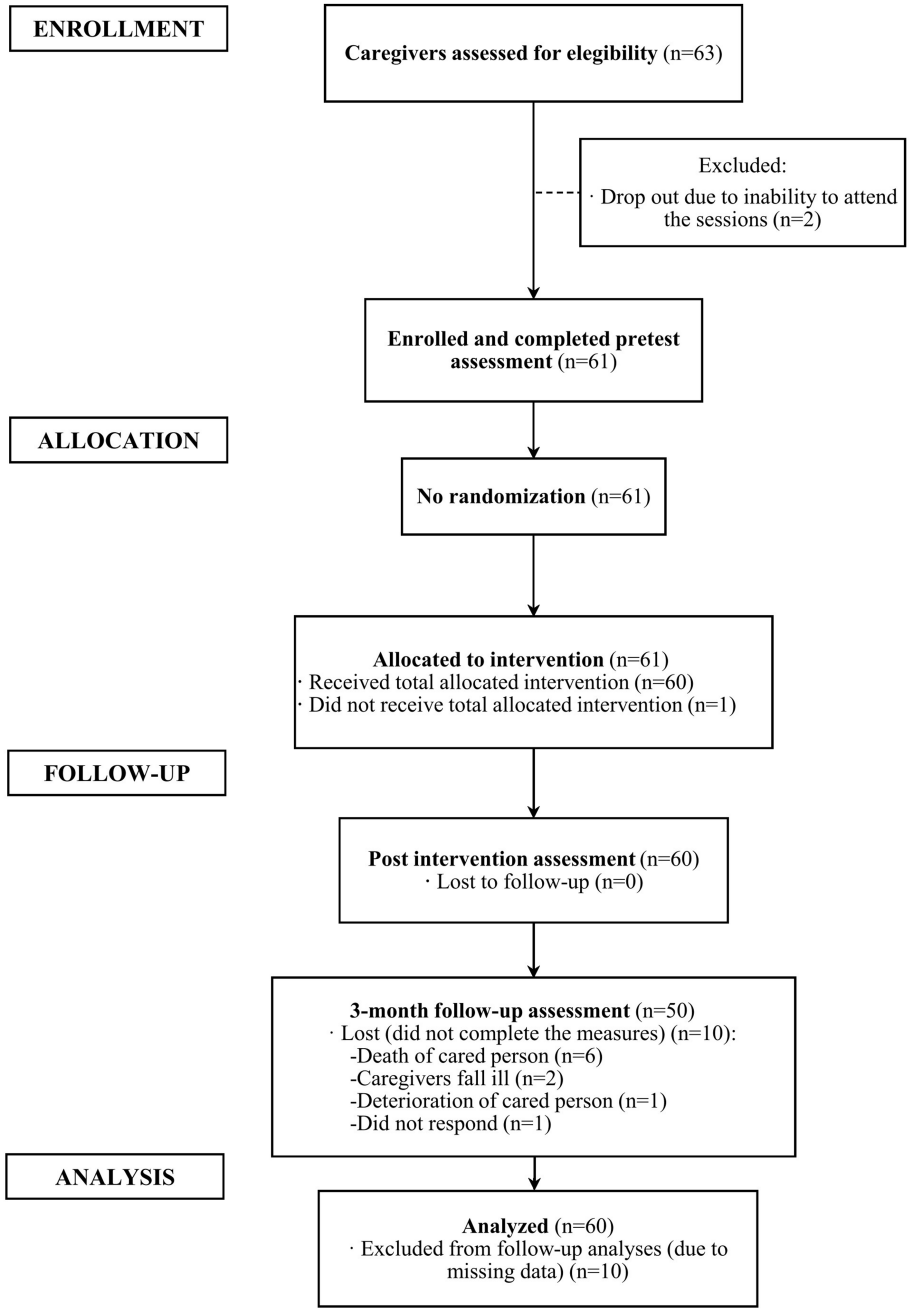
CONSORT flowchart of the participants.

A total of 63 caregivers expressed interest in participating in the music therapy program, completing the pre-intervention assessment. A total of 61 people participated. In total, 11 10-session SGMT programs were conducted with 4–7 people in each group. After one participant abandoned the intervention, the final sample consisted of 60 participants in the pre-assessment and post-assessment phases and 50 participants in the follow-up assessment phase. Figure 2 presents the study flow as per the Consolidated Standards of Reporting Trials (CONSORT) (Bennett, 2005).

In relation to the sociodemographic variables, the intervention group of 60 caregivers included 54 women (90%) and 6 men (10%), aged between 42 and 86 years (66.27 ± 9.827). In relation to kinship, the caregivers were 27 sons/daughters, 26 spouses/partners, 4 siblings, and 3 other types of relationship. Table 1 shows the sociodemographic of the 60 caregivers who participated in this study.

**Table 1:** Sociodemographic characteristics of the caregivers.

**Sociodemographic variables**		***N* (%)** ***n* = 60**
Marital status	Married	40 (66.7%)
	Living as a couple	5 (8.3%)
	Divorced	3 (5%)
	Separated	2 (3.3%)
	Single	6 (10%)
	Widow/er	4 (6.7%)
Educational level*	Less than basic	8 (13.3%)
	Basic	28 (46.7%)
	Intermediate	16 (26.7%)
	Advanced	8 (13.3%)
Occupation	Employed	11 (18.3%)
	Unemployed	7 (11.7%)
	Retired	26 (43.3%)
	Housework	13 (21.7%)
	Other	3 (5%)
Urbanity level	Urban	25 (41.7%)
	Intermediate	26 (43.3%)
	Rural	9 (15%)
Children	Yes	50 (83.3%)
	No	10 (16.7%)

*Reported as the international standard classification of education (UNESCO Institute for Statistics, 2012).

### Instruments

#### Sociodemographic, caregiving data, and experience of SGMT

A 35-item *ad hoc* questionnaire was used to collect the participants’ sociodemographic and caregiving data. Sociodemographic items included age, gender, marital status, academic level, occupation, urbanity level, and having children and caregiving context (relation to EPD). Caregivers’ satisfaction and experience of participating in the sessions were examined via an open-ended question used in a previous music therapy oncology study, which included participants who were visiting patients in a cancer hospital: “Some people say music therapy does not do much for them at all, while other people say that music therapy helps them a great deal. Can you please describe what music therapy did for you during your process?” (O’Callaghan and McDermott, 2004).

#### Caregiver’s overload

Zarit Burden Interview – ZBI (Zarit et al., 1980) was used to assess the level of subjective burden experienced by informal caregivers, providing care to patients with various medical conditions. The instrument consists of 22 items, that cover different aspects of caregiver burden, rated on a five-point Likert scale, where higher scores indicate greater burnout. The Spanish version has been shown to have a good internal consistency (Cronbach’s alpha 0.90) (Ramírez et al., 2008). A Cronbach’s alpha coefficient of 0.88 was obtained in this study.

#### Health-related quality of life (HRQoL)

The Short Form 36 (SF-36) (Ware, 1992) is a questionnaire which is designed to assess individual’s perception of their overall health and well-being across various physical and mental health domains. It measures HRQoL in both clinical practice and research settings, and it consists of 36 items, each of them scored on Likert-type scales with response options ranging from 2 to 6 points, depending on the specific item. It presents eight multi-item scales, which are further categorized into two summary measures: Physical Component Summary (PCS) and Mental Component Summary (MCS). The eight scales are: physical functioning, role limitations due to physical problems (role-physical), bodily pain, general health, vitality, social functioning, role limitations due to emotional problems (role-emotional), and mental health. Higher scores on each scale and summary measure indicate better HRQoL. In relation to the psychometric properties of the Spanish version of SF-36, a study reported good internal consistency, with Cronbach’s alpha between 0.70 and 0.90 in all dimensions of the SF-36 (Ayuso-Mateos et al., 1999). In this study, Cronbach’s alpha coefficient was 0.93.

#### Anxiety

The State–Trait Anxiety Inventory (STAI) (Spielberger, 1970) has two subscales, and each subscale has 20 items. The first subscale measures State Anxiety (S-STAI) which assesses how an individual feels at the moment. The second subscale evaluates Trait Anxiety (T-STAI) which indicates how one feels generally. Item scores range from 0 to 3 points, and the total score ranges from 0 to 60, with higher scores indicating greater anxiety. The Spanish version of the STAI demonstrated good psychometric properties, with Cronbach’s alpha reliability of 0.90 for T-STAI and 0.94 for S-STAI (Guillén-Riquelme and Buela-Casal, 2011). In the current study, Cronbach’s alpha coefficient was 0.91 for T-STAI and 0.95 for S-STAI.

#### Depression

Beck’s Depression Inventory-II (BDI-II) (Beck, 1996) is a 21-item self-report scale that is designed to detect and assess the severity of depression. Each item is rated on a four-point Likert-type scale ranging from 0 to 3, based on the severity over the last 2 weeks. The total score ranges from 0 to 63, with higher scores indicating more severe depressive symptoms. The Spanish psychometric properties show Cronbach’s alpha coefficient of 0.89 (Sanz et al., 2003). The present study obtained Cronbach’s alpha coefficient of 0.90.

#### Spirituality

The instrument World Health Organization Quality of Life about Spirituality, Religiousness, and Personal Beliefs (WHOQOL-SRPB) (WHOQOL SRPB Group, 2002) consists of 32 items. This assessment tool evaluates eight facets of Spirituality, Religiousness, and Personal Beliefs (SRPB) as follows: (a) connectedness to a spiritual being or force; (b) meaning of life; (c) awe; (d) wholeness and integration; (e) spiritual strength; (f) inner peace/serenity/harmony; (g) hope and optimism; and (h) faith. Each item is answered by a five-point Likert scale, ranging from 1 (“not at all”) to 5 (“completely”), where higher scores indicate greater use of that domain of spirituality. The questionnaire design allows it to be administered to individuals with differing ranges of spiritual, religious, and/or personal beliefs. The Spanish version of the questionnaire has presented a good internal consistency with Cronbach’s alpha coefficient of 0.90 (Lucas-Carrasco, 2012). In this study, de Cronbach’s alpha coefficient was 0.97.

#### Cope strategies

The Brief-COPE (Carver, 1997) is a 28-item self-reported questionnaire which is developed to assess different forms of stress response. It consists of 14 subscales of 2 items each, where the response is with an ordinal scale with four alternatives (from 0 to 3), ranging from “I never do this” to “I always do this.” In this case, internal consistency analysis is not recommended for two-item subscales (Morán et al., 2010).

### Procedure

#### Data collection

Before data collection, the University of Deusto Research Ethics Committee approved the research (Ref: ETK-42/20–21), which was conducted in accordance with the Declaration of Helsinki. The trial was registered in clinicaltrials.gov (NCT06028815). Eight different social and health services offered to caregivers of EPD in Bizkaia, Spain, were contacted and asked if the first author could describe the project to the psychologists of the service. All centers agreed that the first author could try and recruit participants at their service by providing an informative talk about the project to the caregivers. Those who agreed to participate signed informed consent forms and provided contact details to the first author. The groups were formed by convenience, according to the number of people interested in each of the services. The reference criteria used to define the groups were that there should be a minimum of 4 people and a maximum of 7 people per group. If there were more than 7 people interested in a service, 2 groups were formed. Once the groups were established, each caregiver received the initial assessment from the first author via email. This assessment involved a self-administered survey conducted through the “Qualtrics” software, a virtual platform accessed using a personal link. This assessment included sociodemographic and caregiving data questions and all earlier mentioned measurement instruments. The contact details of the researcher were provided in the informed consent form in case of any questions arose during the completion of the survey. The anticipated pre-intervention survey completion duration was approximately 1 h. Caregivers then proceeded to begin the 10-session intervention. After 1 week of the end of the intervention, they were invited to complete the same survey again, with an additional open-ended satisfaction question in which they were asked to write about their experience of the SGMT intervention. After 3 months of the second intervention period, the participating caregivers were contacted for the last assessment, which again used the same survey format, excluding the experience of SGMT question.

#### SGMT program intervention

The intervention was carried out in a small group format (4–7 people), with a weekly frequency and a duration of approximately 1 h per session. A total of 10 sessions were held with each group. Although the intervention was carried out in different centers, all of them had a room with similar characteristics, with low reverberation and good ventilation and lighting. In addition, to include the instruments and chairs to sit on, the rooms were practically empty and without decoration. Every group session was conducted by a trained music therapist who was also a trained psychologist. All groups received the same intervention protocol, with therapeutic songwriting being the main technique used. Other music therapy techniques were used throughout the sessions in order to prepare for or close the songwriting activity. These secondary techniques were clinical improvisation (Wigram, 2004) and receptive techniques (e.g., relaxation, listening, and discussion of meaningful songs, and listening for image evocation) (Grocke and Wigram, 2011).

The SGMT program intervention was based on two theoretical models: Fiorini, the “creating psyche” model (Fiorini, 2007) and the Priestley “Analytical oriented music therapy” (AOM) model (Wigram et al., 2002). The Fiorini model represents the processes of this SGMT intervention group, where a safe space of “the possible” has been offered, potentiating arising inner conflicts and movement to personal growth. This model was reflected through the different songwriting processes, where we: (a) started from “the given” (personal or group problematic situation or known song); (b) faced the task of songwriting that could be regarded as “impossible” with blockages and emotions such as fear, uncertainty, and insecurity; and (c) created a real and tangible “possible” which was the composition itself, offering caregivers the experience of managing to overcome “the impossible” and experience personal growth.

The program was also based on AOM, an extension of what was previously known as Analytical Music Therapy. However, this new perspective is not only based on psychoanalytical theories but is also based on communication theories and psychosocial components that are involved in the development of an individual’s personality. AOM involves periods of verbal and musical interaction, seeking the balance between verbal and musical communication. Although this is an improvisation model, in this program intervention we adapted it to the use of songwriting, which also combined language and music. Furthermore, this model is particularly applicable to the SGMT program intervention, since the target population is verbal, as they are adult-elderly people who have been communicating verbally for 50–60 years and do not have cognitive difficulties. The music therapeutic process itself will consist of transferring the main communication from verbal to musical and from music in therapy to music as therapy. Songwriting encompasses verbal processing and communication of important issues as lyrics are generated, and musical expression and communication as music creation can symbolically express significant intrapsychic phenomena (Wigram et al., 2002). The objectives and procedure of the SGMT program, as presented in Table 2, were divided into three different phases: Phase 1—“Getting to know each other”; Phase 2—“Sharing and Creating”; and Phase 3—“Saying goodbye.” Each phase has specific therapeutic objectives, which are achieved by using the main technique (songwriting) as a facilitator. The therapeutic process that emerged was understood as a dynamic and sequential procedure, making it possible to reduce the directionality and structure of the sessions as the process progressed. Considering that one of the strengths of songwriting is its versatility, three methods of songwriting were carried out during the 10 sessions depending on the phase.

**Table 2:** Session content.

**Phase 1 (Sessions 1–3)** **Group parody songwriting**	**Phase 2 (Sessions 4–7)** **Individual parody songwriting**	**Phase 3 (Sessions 8–10)** **Group original songwriting**
Objectives: Detect expectations and needs. To create a link between therapist-caregivers, caregiver-caregiver, caregivers-music. To facilitate communication between the members of the group.	Objectives: To deepen and work on the individual goals of the caregivers. To strengthen and reinforce group cohesion. To facilitate the autonomy of the group.	Objectives: To prepare the group for the closing of the process. To encourage the emotional expression of what has been lived and experienced in order to elaborate on what has been worked on in the sessions.

Phase 1 started with the Group Song Parody, as an introduction to songwriting, where the group chose a traditional song (e.g., “La Bamba” or “La cucaracha”). This first songwriting provided more structure due to the choice of well-known music and the group format, with the music therapist being more directive. The lyrics were composed of words that answered the question “what do you expect from music therapy sessions?

In the second phase, the same method was used but in an individual format, favoring the expression of individual needs, which was supported by the group. The parodied song in the second songwriting was chosen by each caregiver and had to be meaningful for them. To make the Individual Song Parody, they had to answer two questions: “To whom do you want to write?” and “what do you want to say to that person?”

Finally, when the group was already cohesive, it was possible to use the Group’s Original Songwriting, being completely autonomous and able to create without structure or direction from the music therapist. In this case, the music therapist offered them a known structure (ABABCB form), where A is verse, B is the chorus, and C is a bridge. To create the music, caregivers chose instruments and musical parameters (such as pitch, intensity, and timbre). With this information, the music therapist created a base with the guitar (Baker, 2015).

In relation to the music performed during the songwriting in group ones (1 and 3), all of the caregivers sang and played instruments once the songwriting was composed. Regarding the second songwriting, the caregiver who shared the individual composition with the group decided the role of the music (putting the parodied song, singing a cappella, and with or without instruments) and how the participation of the group members would proceed (singing with the caregiver, playing instruments, or just listening).

### Statistical analyses

#### Quantitative analyses

Descriptive statistics were used to describe the participants. Statistical Package for the Social Sciences (SPSS) version 27 program was used to perform analyses on the quantifiable survey data. The continuous variables were described by mean and standard deviation, and the categorical variables were described by frequency and percentage. Regarding the clinical variables, the global scores were calculated. For the SF-36, the syntax provided by the authors of the instrument was applied. The adjustment to the normal distribution of the sample was analyzed for each of the variables studied using the Kolmogorov–Smirnov test. Most variables did not have a normal distribution because the coefficient (K-S) was significant (*p* > 0.05). To verify the change between the pre-test and post-test, 3 months of follow-up measures of the non-parametric test of Wilcoxon’s difference in medians was used, setting a value of p of <0.05 when statistically significant differences occurred between these measures.

#### Qualitative analyses

Open-ended survey response data were written by participants in Spanish and translated by the bilingual (Spanish and English) first author into English. A second professional, an expert in the English language and an experienced qualitative researcher, reviewed and confirmed the translation. The main reasons for this translation were that both of the researchers who participated in the qualitative inter-rate reliability procedures spoke English while only one researcher spoke Spanish and the quotes of the caregivers could be included as part of the results in this English language journal. Thematic analysis was conducted on this data using selected strategies from grounded theory, including comparative, iterative, and predominantly inductive analysis. The “Strauss” approach supports selected grounded theory procedures to generate themes (Strauss and Corbin, 1990). A qualitative inter-rater reliability procedure was also employed, wherein two researchers were involved in the data analysis (Kitto et al., 2008). Initial data analysis involved the first author (PPN) creating and applying a code (label) or codes that characterized the meaning of each participant’s response. CO (second author), an experienced qualitative researcher, then examined all the data and PPN’s coding. PPN and CO discussed coding inconsistencies until mutually agreed on the set of codes. Coded segments were compared by PPN with other coded segments to determine similarities and differences. Similar coded segments were grouped under researcher-created categories (researcher-created labels representing comparable code groups). CO then examined PPN’s categories, and the two researchers continued to discuss comparable and different interpretations of the categories until reaching an agreement. PPN then compared and contrasted the categories, and similar categories were grouped together under researcher-created themes (labels representing comparable category and code groups). CO then examined PPN’s themes, and the two researchers continued to discuss comparable and different interpretations of the themes until reaching an agreement on the final representation of the findings. ATLAS.ti 22 (PPN) and ATLAS.ti 9 (CO) qualitative data management software for Windows was also employed.

## Results

### Quantitative results

First, a Wilcoxon analysis was conducted in order to define pre-differences and immediate post-differences. Statistically significant outcomes are shown in Table 3. A moderate effect size was observed in two total scores (STAI-T and BDI-II) and three sub-scores. Although all other analyses indicated no statistically significant differences between pre-measures and immediate post-measures, the post-test means were higher than the pre-test means on the other subscales of SF-36 and SRPB and lower on STAI-S and Zarit (Supplementary Table S1).

**Table 3:** Significant results between the overall pre-post intervention measures.

**Scale**	**Pre (*n* = 60)** **M (SD)**	**Post (*n* = 60)** **M (SD)**	***Z***	***p***	***r***
**STAI-T**	28.68 (10.37)	25.95 (9.14)	−3.258	0.001	−0.297**
**BDI-II**	14.10 (9.15)	11.9 (8.4)	−2.562	0.010	−0.234*
**WHOQOL-SRPB**					
Inner Peace	2.68 (0.75)	2.88 (0.79)	2.220	0.026	0.203*
**SF-36**					
Social functioning	60.42 (25.54)	68.33 (25.67)	−2.364	0.018	−0.216*
Mental health	52.40 (12.90)	55.47 (11.84)	−2.072	0.038	−0.189*

M, mean; SD, standard deviation; *p < 0.05, **p < 0.01; *r*, correlation biserial.

Another analysis was made to determine if there were differences between the immediate post-measures and the 3-month follow-up measures. The statistically significant difference was decreased trait anxiety (*Z* = -2.035, *p* = 0.042, *r* = −0.19).

The last analysis carried out was between the pre-measures and 3-month follow-up measures, and statistically significant outcomes are shown in Table 4. A moderate effect size was observed in four total scores (STAI-T, STAI-S, BDI-II, and Zarit) and five sub-scores. No more significant differences were found for the other outcomes, but the means were higher on other subscales of SF-36 and SRPB (Supplementary Table S2).

**Table 4:** Significant results between the overall pre-intervention and follow-up measures.

**Scale**	**Pre (*n* = 60)** **M (SD)**	**Follow-up (*n* = 50)** **M (SD)**	***Z***	***p***	***r***
**STAI-S**	29.58 (11.75)	26.06 (12.51)	−2.633	0.008	−0.24*
**STAI-T**	28.68 (10.37)	24.52 (10.25)	−3.258	0.001	−0.30**
**BDI-II**	14.10 (9.15)	12.34 (9.68)	−2.275	0.023	−0.21*
**ZARIT**	59.68 (14.45)	56.06 (16.23)	−2.077	0.038	−0.19*
**WHOQOL-SRPB**					
Whole	2.8 (0.79)	3.02 (0.80)	−2.196	0.028	−0.20*
Inner Peace	2.68 (0.75)	2.92 (0.78)	−2.557	0.011	−0.23*
**SF-36**					
Standarized mental component	37.18(12.17)	39.32 (11.87)	−1.955	0.051	−0.18*
Social functioning	60.42(25.54)	66.00 (25.76)	−2.129	0.033	−0.19*
Bodily pain	50.92(25.38)	58.52 (24.89)	−2.010	0.044	−0.18*

M, mean; SD, standard deviation; *p < 0.05, **p < 0.01; *r*, correlation biserial.

### Qualitative results

**Figure 3:**
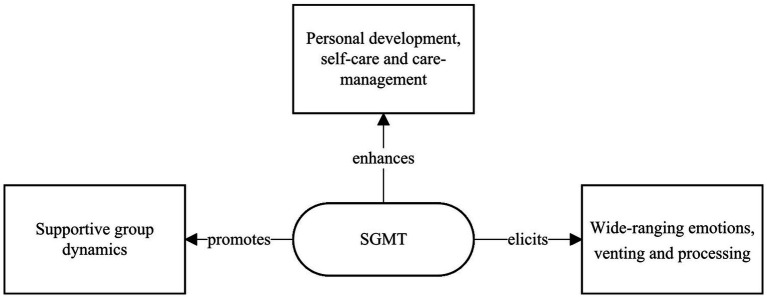
SGMT experience main themes.

Three themes were generated from the thematic analysis. Caregivers reported that SGMT participation can enhance personal growth, bring out and enable work on emotions, and promote helpful interpersonal dynamics (Figure 3). Clarification of the themes is illustrated with participant quotes. To differentiate the quotes of the participants while maintaining confidentiality, an alphanumeric code consisting of their initials and the response number has been used.

#### Theme 1. SGMT can enhance caregivers’ personal development, self-care, and care management

**Self-awareness, personal growth, and self-care:** Many caregivers described how the SGMT sessions enabled their personal improvement and self-development and/or provided opportunities for self-focus and self-care. ALF-5 explained, “for me personally it made me think about myself, give myself (that) permission.” Some participants emphasized how SGMT helped them to understand the relevance of dedicating time to themselves: Comments included how the sessions taught caregivers “to take care of myself” (MGV-29), “think about my needs” (BFS-10), and to experience the “luxury” of “dedicat(ing) an hour for me” (BQA-26). Several participants also explained how they worked on personal growth and developed increased self-awareness in the sessions. Statements included how SGMT “made me look inside myself” (BFS-10; NPC-48), “helped me to get to know myself” (JAO-3), find “myself” (EGG-51; BBZ-8), and “connect with myself” (CFO-39; RMN-7).

**Coping strategies:** Some caregivers described how their SGMT experience helped them to develop strategies, which enabled them to manage in their care situations. Individual participants said that SGMT helped them to “accept a little more your hard reality” (MCC-35), learn a “tool to face a solution” (JHB-18), “overcoming” (EGG-51), and “Focus and live in the moment. Live my circumstances from another point of view. Find a place in me to escape from the situation and be able to move forward” (MEA-15). Several participants also said that SGMT helped them to focus on something other than their caregiver role. For example, MDGB-55 experienced “escape and disconnection during the sessions” (MDGB-55). Other caregivers wrote that SGMT allowed them to “concentrate and forget about everything else” (RAU-2), feel “distracted … from my worries during and after the sessions” (CBLL-19), “escape from day-to-day issues” (JAO-3), and “disconnect from the daily routine” (FST-53).

#### Theme 2. SGMT can elicit caregivers’ wide-ranging emotions and enable helpful venting and processing

**Bringing out emotions:** SGMT elicited emotions in many caregivers, with individuals describing how it “brings out emotions” (JDP-40), enabled “awakening emotions” (VGJ-45), and made them “feel better” (CCH-38, JDP-40, and FGA-60) and “good” (AGT-17). Caregivers described wide-ranging emotions experienced in SGMT, including that sessions were “helping me to relax” (PML-23) and enabling feelings of “inner blocks and sadness” (MAD-24) and expression of “pain” (MCC-35), “peace, joy (and) emotion” (MLG-34). BHA-33 explained, “It has been entertaining, pleasant, fun and that’s enough for me.” These emotions and feelings could be present both during the sessions and the following days: “(SGMT sessions were) an appointment that made me feel better, calmer for the rest of the day and the following days.” (CCH-38).

**Expressing and processing personal emotions:** In addition to eliciting emotions, SGMT may help some caregivers express previously unexpressed emotions. Individual caregivers also reported that SGMT helped them through “get(ting) my feelings out” (CFO-39), “expressing myself” (MJFCH-42), “bring(ing) out my feelings” (BFSNB-10, RRG-13, MLG-34, and MCC-35), “venting” (MTCG-16, NPC-48, and CCH-38), and producing a “sense of relief” (JVM-22). SGMT also provided some participants with an opportunity to approach and identify significant personal emotions. For example, SMR-9 said that SGMT was “stirring my inner self,” and ADD-25 stated that “it helped to process my feelings.” This could include significant issues from their past, with individual caregivers commenting that SGMT sessions allowed one to “take things out of the past” (MTCG-16) and another to “look inside myself and bring out many feelings that I had not stopped to feel or see before.” (NPC-48).

#### Theme 3. The songwriting music therapy group’s interpersonal dynamics support caregivers

**Cohesiveness promoting sharing:** SGMT could facilitate the creation of a cohesive group, providing a setting conducive for helpful and diverse interpersonal dynamics. The SGMT was described as an opportunity “to meet great people” (MGV-29) and “relate to people I did not know at all” (MRC-31). Individual caregivers explained that SGMT helped them to experience a “union with companions” (MCC-35; CCH-38) and to “connect with companions” (RMN-7; SMR-9). Statements from several participants indicated the importance of the group process. JVM-22 wrote that “you (can) count on the group”, and ERS-41 wrote that “between all of us we have built something beautiful.” SGMT could promote the teamwork, with BCR-6 reporting that “doing it in a group and participating in the lyrics and music was interesting.” IGL-57 also explained that “it has helped me to work as a team (member).”

The cohesive group helped some caregivers to feel comfortable to discuss and share their situations, problems, and experiences. Quotes included how SGMT was a safe place “supporting me to see my real situation…helping me to talk about the problem as a caregiver” (PML-23) and “it helped me to open up more… and be with other people” (MPPB-46). NPC-48 highlighted that the group could help members to manage their emotions, stating, “sharing personal experiences with the group is enriching and helps to vent.”

**Group identification and support:** Some caregivers indicated that an important factor in forming this connected group was that it comprised people who were going through similar situations. Comments included that, “it helped me to process my feelings and to be able to share them with other people who know what we are talking about” (AAD-25), “it brought me closer to people who are in a similar situation to mine” (MMES-32), and “they were joyful moments and shared with companions who understand the situation well” (IOI-43). Perceiving the other caregivers as equals could promote the feeling of, “I am not the only one going through adverse moments” (BBZ-8) and “I am not the only one. You see other people’s problems” (MJFCH-42). VGL-45 reported that she/he “identified with the others.” The SGMT could allow caregivers to compare their different situations and consider helpful different perspectives. MBGM-47 wrote that SGMT helped her “to see that I was not alone in the hard work of caregiving and that there were people in the group with more difficult problems than mine” (MBGM-47).

Sharing similar backgrounds could also help some caregivers “to feel listened to” (RRG-13, MTCG-16, RGM-36, and JMM-37) and heard. Individual participants felt that the group “understood” (RRG-13) and “supported” (RGM-36) them and “accepted and loved” them (JMM-37). As CCH-38 said, SGMT allowed caregivers “to feel understood and supported by others to the point of feeling united with others”. Finally, MIPP-44 asserted that the group moderator was also important in enabling the helpful group process, explaining that “the group and the person who moderated the group have been a very strong support. It has taught us to share our problems and solve them together. It has been a good teaching (therapy) through music.”

## Discussion

The physical and psychological symptomatology associated with informal caring can have a serious impact on the QoL of caregivers (Schulz et al., 1990, 1995, 1997; Pot et al., 1997; Pinquart and Sörensen, 2003; Verbakel et al., 2016; Espinoza-Salgado et al., 2017; Bom et al., 2018; Choi and Seo, 2019; Perpiñá-Galvañ et al., 2019; Oechsle et al., 2020). These consequences of caring for health must be addressed from a multidisciplinary approach, considering the needs of the informal caregiver. Many therapeutic disciplines can be used to improve their well-being (Warth et al., 2019; Ahn et al., 2020), with music therapy being a very powerful tool, demonstrating effectiveness in improving the QoL of informal caregivers (Clair et al., 1993; Clair, 2002; Brotons and Marti, 2003; Magill, 2009; Choi, 2010; Kim and Dvorak, 2018; Potvin et al., 2018; Gok Ugur et al., 2019; García-Valverde et al., 2020; Valero-Cantero et al., 2020; Wilhelm and Wilhelm, 2020; Brault and Vaillancourt, 2022; Denk, 2023; Whitford et al., 2023).

The purpose of the current study was to describe the experiences of informal caregivers by a mixed method approach, participating in an SGMT intervention. The findings of this research could show that participation in the SGMT program intervention improved the QoL of the informal caregivers. In particular, it demonstrated changes in informal caregivers’ emotional and social variables and their in-depth personal work.

In relation to the psychological symptoms, quantitative findings showed that trait anxiety and depression were significantly lower immediately after finishing the SGMT intervention. Additionally, the inner peace, social function, and mental health scores also showed improvements. These changes were sustained over time, which were indicated by non-significant differences when 3 months of follow-up scores were compared with the immediate post scores, except for the trait anxiety, which was even lower. At the 3-month follow-up, additional variables (including state anxiety, burden, bodily pain, and the spiritual subscale “whole”) which were unchanged immediately after SGMT showed the same magnitude of improvement as that described for trait anxiety and depression. These findings might suggest that several of the positive effects of a 10-week SGMT program only emerge for 3 months following cessation of the program, requiring more time to develop and put into practice what was worked on in the sessions.

Despite the fact that without the control group, the variable promoting these changes cannot be determined, the outcomes of SGMT with informal caregivers are consistent with earlier research findings that showed positive change in QoL. Specifically, these changes are reported in trait anxiety, depression, spirituality, mental health, and social functioning after music therapy treatment (Clair et al., 1993; Brotons and Marti, 2003; Choi, 2010; Baker et al., 2018; García-Valverde et al., 2020; Denk, 2023). Our investigation confirmed that these benefits, which are potentially a result of the SGMT, were maintained for 3 months following the intervention. Our finding extends the evidence which demonstrates sustained caregiver improvements, following a series of music therapy sessions. Positive long-term results were found in only a few other studies with informal caregivers of adult people (Brotons and Marti, 2003; Whitford et al., 2023).

Additionally, three thematic findings in this research reinforced the value of SGMT for participants. Through the deep analysis of the experience of the informal caregivers participating in SGMT, the findings were that SGMT enhanced personal growth and self-care; brought out and enabled work on emotions, and promoted helpful interpersonal dynamics.

In this study, informal caregivers described how SGMT improved their personal growth, self-care, and enhanced self-awareness. Sessions also helped them to learn strategies that supported their caring roles and focus on something other than caring, working on themselves and their self-care. Another highlighted theme was the emotional dimension. Caregivers said that SGMT allowed them to express and process their feelings. The finding about interpersonal dynamics, whereby caregivers described the importance of feeling cohesively supported by group members who were going through similar situations, is strongly related to certain therapeutic factors described by Yalom that are reflected in group psychotherapy such as “universality” and “cohesion”(Vinogradov and Yalom, 1996).

Comparing these findings, the outcomes are consistent with previous studies conducted with informal caregivers. A recent study using thematic analysis to assess informal caregivers’ experiences of participating in a songwriting group found that caregivers reported personal improvement, exploration and expression of emotions, group identification, connection, and mutual support (García-Valverde et al., 2022). Another study found that group songwriting enabled caregivers to experience personal growth, insights about coping, connection, and group identity (Baker et al., 2018). Further literature also confirms our study findings related to music therapy’s impact on improving therapeutic insight, positive emotions, and group cohesiveness (Klein and Silverman, 2012).

**Figure 4:**
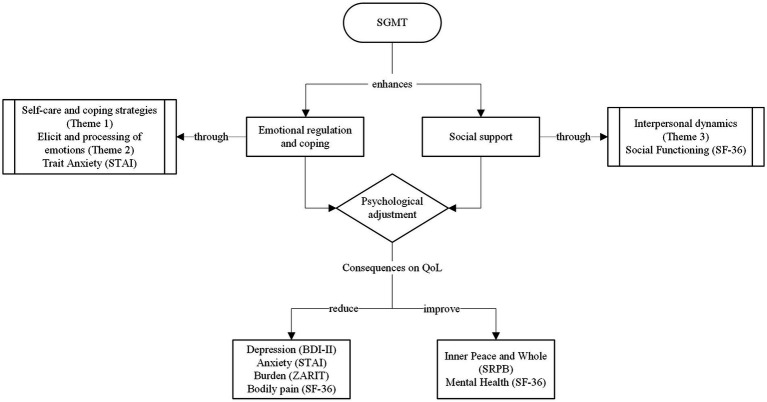
Effects of SGMT on informal caregivers: a model informed by the converged findings.

As a result of the mixed methodology, the converged findings that enable consideration of multidimensionality of the QoL model can be established to illuminate understanding of the effects of SGMT on caregivers (Figure 4).

The findings show how SGMT enabled work on self-regulation and coping in the way that caregivers understood and managed the care situation and emerging emotions. This statement can be observed in two of the themes from the thematic analysis on the importance of self-care and care management (theme 1) and on elicited and processed emotions (theme 2). Furthermore, the fact that the strongest change in caregivers’ QoL was in the reduction of trait anxiety, understood as a stable personality tendency, shows the in-depth work done on ameliorating the way in which the caregivers perceive situations as threatening, such as in this case caring for a family member (Endler and Kocovski, 2001).

SGMT also enhanced social support. This improvement in the social dimension was reflected in two aspects: First, in the third theme about interpersonal dynamics which showed that SGMT was a place where caregivers felt supported and identified with others, and second, in the change related to the social dimension (in one of the domains of the SF-36, social functioning) where change was maintained over 3-months of the intervention.

Considering this psychosocial work carried out in the sessions, it can be concluded that through the SGMT, caregivers can achieve an improved psychological adjustment. It is understood that social support and functional coping may be protective factors for caregiver overload symptomatology and predictors of QoL (Blankfeld and Holahan, 1999; Di Mattei et al., 2008; Iavarone et al., 2014; Serra et al., 2018; Leung et al., 2020). Thus, it could be assumed that the effects of SGMT have consequences for caregivers’ QoL, such as improved spirituality and reduced depression. Changes were found between pre-SGMT and 3-month post-SGMT assessments for a number of outcomes, including caregiving overload, bodily pain (which may be related to caregiving itself), and state anxiety, indicating a slow onset of treatment effects. Through the development of this model of effects, it is understood that the caregivers’ in-depth work on how they understood and managed caregiving, with the support of their peers, led to decreases in certain symptomatology over time, such as state anxiety. Therefore, in the SGMT intervention, by working not only on transitory changes but also on profound internal ones, caregivers can acquire strategies and tools that can be applied over time, leading to a reduction in overload (Lee et al., 2022).

The major discrepancy in converging the data lies in the lack of differences observed in coping strategies in the quantitative data, with no significant differences in any of the subscales between any of the assessment times. However, in the qualitative data, it can be observed how the SGMT has facilitated the caregivers’ personal growth through learning and applying tools to manage the caregiving situation. These differences may be due to the fact that coping strategies are an ingrained part of the personality and in this case, questionnaires, such as the one used, are not very sensitive to small changes because each dimension is composed of just two items (Morán et al., 2010). However, qualitative data have allowed us to explore this aspect more deeply by capturing the experiences of the participants, considering that mixed methods methodology can highlight aspects that may otherwise remain overlooked because they are not sufficiently represented by numerical data.

### Limitations and future research

Interpretations of the findings reported in this study need to be made in relation to the limitations of the study. First, there was no comparison in the control group, thus it cannot be concluded that the quantitative findings are because of the effect of the intervention. Incorporating a comparative group would have allowed the assessment of temporal change unrelated to treatment. Second, the same person (first author) conducted multiple roles in the research, which could have biased the results. The first author was the music therapist who conducted the SGMT sessions, and one of the researchers involved in data analysis, especially in the process of thematic analysis. Qualitative data analysis is an interpretive process; however, the inter-rater reliability process should have increased the trustworthiness of the qualitative findings. Third, there was a gender imbalance in the sample (more women than men), which reflects a greater number of women caregivers in our community (Cueli et al., 2018). Nevertheless, it could be a problem that the small sample size of male participants may result in the findings that do not reflect males’ responses to SGMT, limiting transferability (del Río et al., 2017). Another limitation of this study is that the sample size could be bigger in order to reduce statistical issues such as the Type II Error. Finally, the assessment protocol may have been too long for this group of participants, many of whom were partners of older people. This could have affected the reliability of the results. However, we hope that the present study can serve as a resource for future research, guiding the focus of assessments toward the pertinent study variables, particularly addressing the needs of informal caregivers.

Considering the findings and limitations of this study, suggestions for future investigations on the role of psychosocial interventions for informal caregivers of EPD can be made. First, it could be interesting to examine the caregiver participants’ pre-existing psychosocial states and compare them with a normal population to elucidate their distress levels and the types of challenges they may be experiencing in their roles (if any). Second, future research could include the further use of qualitative methodology to deeply investigate informal caregivers’ situations and how they feel about this role. This information could be gathered through the use of interviews and the lyric analysis of songs composed of participants during SGMT.

## Conclusion

The use of music therapy with caregivers of EPD is a novel line of research, and this study reaffirms the benefits reported in other studies with caregivers from other populations. The improvement in caregivers’ QoL, found in this mixed method study, highlights the importance of including psychosocial interventions, such as music therapy, in support services for informal caregivers of EPD. Specifically, this study suggests that SGMT can promote caregivers’ in-depth self-care and enhance their social and emotional functioning and psychological adjustment. The importance of these variables as possible predictors of QoL should be taken into account, in order to focus on interventions on working on them with informal caregivers in group formats. In addition, this research has demonstrated the relevance of including the subjective experiences of people participating in intervention studies, in order to access outcomes not captured by numerical data. It is hoped that the findings of this study will also provoke the scientific community’s interest in further music therapy research with caregivers of EPD using a controlled design.

## Data availability statement

The raw data supporting the conclusions of this article will be made available by the authors, without undue reservation.

## Ethics statement

The studies involving humans were approved by the Research Ethics Committee of the University of Deusto. The studies were conducted in accordance with the local legislation and institutional requirements. The participants provided their written informed consent to participate in this study.

## Author contributions

PP-N: Conceptualization, Data curation, Formal analysis, Investigation, Methodology, Software, Writing – original draft, Writing – review & editing. CO’C: Formal analysis, Investigation, Methodology, Supervision, Writing – review & editing. JL-P: Data curation, Writing – review & editing. ARL: Data curation, Formal analysis, Investigation, Writing – review & editing. AAR: Writing – review & editing, Methodology, Investigation and Resources. IA: Conceptualization, Data curation, Formal analysis, Investigation, Software, Supervision, Writing – review & editing.
