# Supplementary material for: Songwriting Group Music Therapy to promote psychological adjustment in informal caregivers of elderly people with dependency: a mixed methods study
Source: Front Psychol. 2024 Mar 6;15:1334875. doi: 10.3389/fpsyg.2024.1334875 (PMC10953728; doi:10.3389/fpsyg.2024.1334875)
Supplement: Supplementary file 1 [file Table_1.pdf]

Supplementary table 1

Pre and post non-significative measures

Scale	Pre (n=60) M(SD)	Post (n=60) M(SD)	Z	*p*
**STAI-S**	29.58 (11.75)	27.57 (11.42)	-1.149	.250
**WHOQOL-SRPB**				
Connect	2.18 (0.98)	2.28 (1.08)	-0.946	.344
Meaning	2.95 (0.93)	3.12 (0.83)	-1.663	.096
Awe	3.02 (0.72)	3.01 (0.68)	-0.216	.829
Whole	2.80 (0.79)	2.93 (0.78)	-1.476	.140
Strenght	2.48 (1.02)	2.6 (1.03)	-1.381	.167
Hope	2.97 (0.62)	3.02 (0.74)	-0.650	.516
Faith	2.38 (1.06)	2.52 (1.04)	-1.675	.094
**ZARIT**	59.68 (14.45)	58.13 (14.93)	-1.174	.240
**COPE-28**				
Active Coping	1.81 (0.57)	1.86 (0.54)	-0.685	.493
Planning	1.38 (0.59)	1.53 (0.58)	-1.501	.133
Use of Emotional Support	1.57 (0.69)	1.63 (0.62)	-0.487	.626
Use of Instrumental Support	1.55 (0.61)	1.50 (0.63)	-0.365	.715
Religion	0.93 (0.74)	1.02 (0.82)	-1.261	.207
Positive Reframing	1.15 (0.53)	1.22 (0.68)	-0.828	.407
Acceptance	1.86 (0.56)	1.89 (0.58)	-0.471	.638
Denial	0.55 (0.64)	0.63 (0.71)	-0.611	.541
Humor	0.54 (0.64)	0.52 (0.74)	-0.601	.548
Self-distraction	1.39 (0.70)	1.43 (0.74)	-0.428	.669
Self-blame	0.60 (0.61)	0.58 (0.59)	-0.229	.819
Behavioral Disengagement	0.33 (0.51)	0.26 (0.48)	-1.217	.224
Venting	1.01 (0.63)	1.00 (0.66)	-174	.862
Substance Use	0.08 (0.26)	0.07 (0.23)	-0.707	.480
**SF-36**				
Physical Functioning	74.92 (23.08)	76.09 (21.94)	-336	.737
Role-Physical	48.75 (42.79)	57.50 (41.25)	-1.344	.179
Bodily Pain	50.92 (25.38)	55.13 (22.94)	-1.156	.248
General Health	54.03 (21.92)	54.93 (21.44)	-0.343	.732
Vitality	49.33 (19.50)	52 (18.55)	-1.421	.155
Role-emotional	52.78 (46.05)	54.45 (45.91)	-0.299	.765
Physical Component	44.22 (10.61)	45.60 (9.73)	-0.883	.377
Mental Component	37.18 (12.17)	38.78 (11.09)	-1.156	.248
