# Supplementary material for: Songwriting Group Music Therapy to promote psychological adjustment in informal caregivers of elderly people with dependency: a mixed methods study
Source: Front Psychol. 2024 Mar 6;15:1334875. doi: 10.3389/fpsyg.2024.1334875 (PMC10953728; doi:10.3389/fpsyg.2024.1334875)
Supplement: Supplementary file 2 [file Table_2.pdf]

Supplementary table 2

Pre and follow-up non-significative measures

Scale	Pre (n=60) M(SD)	Follow-Up (n=50) M(SD)	Z	*p*
**WHOQOL-SRPB**				
Connect	2.18 (0.98)	2.22 (1.04)	-0.602	.547
Meaning	2.95 (0.93)	3.11 (0.88)	-1.153	.249
Awe	3.02 (0.72)	3.15 (0.67)	-0.530	.596
Strength	2.48 (1.02)	2.60 (1.02)	-0.129	.897
Hope	2.97 (0.62)	2.99 (0.75)	-0.634	.526
Faith	2.38 (1.06)	2.52 (1.09)	-0.721	.471
**COPE-28**				
Active Coping	1.81 (0.57)	1.76 (0.56)	-0.446	.656
Planning	1.38 (0.59)	1.54 (0.67)	-1.686	.092
Use of Emotional Support	1.57 (0.69)	1.62 (0.59)	-0.527	.598
Use of Instrumental Support	1.55 (0.61)	1.41 (0.61)	-1.396	.163
Religion	0.93 (0.74)	0.95 (0.74)	-0.365	.693
Positive Reframing	1.15 (0.53)	1.27 (0.65)	-1.173	.241
Acceptance	1.86 (0.56)	1.99 (0.70)	-1.382	.167
Denial	0.55 (0.64)	0.48 (0.53)	-0.643	.520
Humor	0.54 (0.64)	0.45 (0.59)	-1.272	.203
Self-distraction	1.39 (0.70)	1.39 (0.64)	-0.066	.948
Self-blame	0.60 (0.61)	0.54 (0.57)	-0.695	.487
Behavioral Disengagement	0.33 (0.51)	0.4 (0.55)	-0.773	.440
Venting	1.02 (0.63)	1.02 (0.64)	-0.243	.808
Substance use	0.08 (0.26)	0.05 (0.21)	-1.518	.129
**SF-36**				
Physical Functioning	74.92 (23.08)	76.30 (23.45)	-0.669	.503
Role-Physical	48.75 (42.79)	55.50 (46.37)	-1.363	.173
General Health	54.03 (21.92)	57.94 (21.37)	-1.599	.110
Vitality	49.33 (19.50)	52.50 (21.72)	-1.081	.280
Role-Emotional	52.78 (46.05)	61.34 (44.85)	-1.692	.091
Mental Health	52.40 (12.90)	54.64 (13.75)	-1.264	.206
Physical Component	44.22 (10.61)	45.75 (10.68)	-0.912	.362
Mental Component	37.18 (12.17)	39.32 (11.87)	-1.955	.051
